# Quantification of HIV-1 RNA Among Men Who Have Sex With Men Using an At-Home Self-Collected Dried Blood Spot Specimen: Feasibility Study

**DOI:** 10.2196/10847

**Published:** 2018-11-01

**Authors:** Sabina Hirshfield, Richard A Teran, Martin J Downing Jr, Mary Ann Chiasson, Hong-Van Tieu, Laura Dize, Charlotte A Gaydos

**Affiliations:** 1 Research and Evaluation Public Health Solutions New York, NY United States; 2 Department of Epidemiology Mailman School of Public Health Columbia University New York, NY United States; 3 Psychology Department Lehman College Bronx, NY United States; 4 Division of Infectious Diseases Department of Medicine Columbia University Medical Center New York, NY United States; 5 Laboratory of Infectious Disease Prevention New York Blood Center New York, NY United States; 6 Division of Infectious Diseases Johns Hopkins University Baltimore, MD United States

**Keywords:** HIV-1, viral load, dried blood spot testing, men who have sex with men

## Abstract

**Background:**

Suboptimal antiretroviral therapy (ART) adherence and disengagement in care present significant public health challenges because of the increased probability of HIV transmission. In the United States, men who have sex with men (MSM) continue to be disproportionately affected by HIV, highlighting a critical need to engage high-risk MSM living with HIV who are not engaged or retained in care.

**Objective:**

The aim of the study was to assess the feasibility of at-home blood self-collection and laboratory quantification of HIV-1 RNA viral load (VL) to report laboratory-based VL outcomes and compare self-reported and laboratory-reported VL

**Methods:**

Between 2016 and 2017, 766 US HIV-positive MSM enrolled in a Web-based behavioral intervention were invited to participate in an at-home dried blood spot (DBS) collection study using HemaSpot-HF kits (Spot On Sciences, Inc, Austin, TX) for laboratory-quantified VL.

**Results:**

Of those invited to participate, 72.3% (554/766) enrolled in the DBS study. Most (79.2%, 439/554) men enrolled reported attempting to collect their blood, 75.5% (418/554) of participants mailed a DBS specimen to the research laboratory, and 60.8% (337/554) had an adequate blood sample for VL testing. Of the 337 specimens tested for VL by the laboratory, 52.5% (177/337) had detectable VL (median: 3508 copies/mL; range: 851-1,202,265 copies/mL). Most men (83.9%, 135/161) who returned a DBS specimen with laboratory-quantified detectable VL self-reported an undetectable VL during their last clinical visit.

**Conclusions:**

Home collection of DBS samples from HIV-positive MSM is feasible and has the potential to support clinical VL monitoring. Discrepant laboratory HIV-1 RNA values and self-reported VL indicate a need to address perceived VL status, especially in the era of treatment as prevention. Most participants were willing to use an at-home DBS kit in the future, signaling an opportunity to engage high-risk MSM in long-term HIV care activities.

## Introduction

### Background

Suboptimal antiretroviral therapy (ART) adherence and intermittent engagement in care present significant public health challenges because of the increased probability of HIV transmission resulting from high HIV-1 RNA viral load (VL) [1-5]. It is critical to assess strategies to monitor VL among individuals living with HIV who are not consistently ART adherent. In the United States, men who have sex with men (MSM) continue to be disproportionately affected by HIV; in 2016, MSM accounted for 66.79% (26,570/39,782) of all HIV diagnoses and 82.69% (26,570/32,131) of diagnoses among men [1]. Among US MSM known to be living with HIV in 2014, 74.07% (265,280/358,151) had received any care, 57.66% (206,523/358,151) were retained in care, and 61.16% (219,043/358,151) of those in care achieved viral suppression, although engagement in care and viral suppression were lowest among younger MSM and black MSM [6,7]. Research and program initiatives have aimed to increase both the number of MSM who are tested and who engage in care after an HIV diagnosis [8-10]. At-home rapid HIV self-testing has provided another option for MSM to be tested, and studies have shown that MSM are willing to self-test rather than use traditional testing sites because of stigma, privacy-related concerns, and the ability to test at any time [11-13]. There is a similar need for VL self-testing or sample collection approaches to be developed for MSM living with HIV that can support traditional HIV clinical care and increase the proportion of virally suppressed MSM living with HIV.

The Mailed-Spot (M-Spot) study assessed the feasibility of home self-collection of dried blood spot (DBS) specimens for laboratory quantification of VL among US white, black, and Hispanic MSM living with HIV who participated in a Web-based behavioral intervention [14]. Because MSM commonly use the Internet and smartphone apps for sexual and health purposes, Web-based and mobile settings provide an opportunity for engagement and obtaining biologic specimens in behavioral research [15-17].

### Study Objectives

We report feasibility and VL outcomes among MSM living with HIV who received a novel DBS collection kit for at-home blood self-collection and laboratory quantification of VL.

## Methods

### Study Overview

MSM participating in Sex Positive! (parent study), a national Web-based behavioral intervention, were invited to take part in the M-Spot study following completion of the original study. The parent study’s protocol has been described previously [14]. Briefly, eligible participants in the parent study were (by self-report) biologically male and identified as a male or genderqueer; aged 18 years or older; white, black, or Hispanic; able to read and respond in English; a US resident; HIV-positive; not virally suppressed (>200 copies/mL) in the past year or reported past-month suboptimal ART adherence [18]; and had condomless anal sex with an HIV-negative or unknown status male partner in the past 6 months.

### Ethics Statement

The institutional review board (IRB) at Public Health Solutions in New York, NY, approved all study procedures. The IRB at Johns Hopkins University in Baltimore, MD, approved all laboratory-related procedures. Participants provided consent by clicking a button at the end of the Web-based consent form to indicate that they had read the consent page and agreed to participate. A Certificate of Confidentiality was obtained from the National Institute of Mental Health to protect the privacy of participants enrolled in this study.

### Participants

For the M-Spot study, men received an email recruitment solicitation within a week of completing the parent study’s 12-month follow-up survey. The email contained a link that redirected them to a brief, secure screening survey. Those ever diagnosed with hemophilia, or who were currently taking anticoagulation medication, were excluded.

### Study Procedures

Consenting participants were mailed a package containing one HemaSpot-HF device (DBS kit; Spot On Sciences, Inc, Austin, TX), collection materials (alcohol prep pads, lancets, gauze pad, and adhesive bandages), an instruction card, and a return envelope with postage. Men read the instruction card or viewed a video that demonstrated how to collect their blood and mail their DBS specimen to the International STD Research Laboratory at Johns Hopkins University.

After self-collecting a DBS specimen, men completed a brief Web-based survey (herein referred to as the *M-Spot survey*), which inquired about the blood collection process; experience using the kit (ie, attempts to use the kit, experience using the lancet, etc); experience with the study materials (ie, did they watch the video, did they understand the instruction card); willingness to use a DBS kit in the future; and engagement in HIV care since the parent study’s 12-month follow-up survey. After collecting their blood specimen and completing the M-Spot survey, men mailed their DBS specimen to the research laboratory.

HemaSpot-HF was developed to address technical issues associated with using traditional filter cards for DBS collection [19]. A protective plastic cartridge minimizes the risk of contamination and contains a desiccant ring to keep the sample free from moisture. Immediately after blood collection, the desiccant allows the kit to be closed for shipment. Upon receipt at the laboratory, DBS specimens were stored for up to 4 months at 4°C before testing. If a DBS kit was half-filled with blood or not filled at all, the sample was deemed untestable. Acceptable samples were tested in batches corresponding to laboratory receipt date.

DBS specimens were placed in an Abbott Master Mix Tube (Abbott Molecular Inc, Des Plaines, IL) containing 1.3 mL of Abbott mSample Preparation system DBS Buffer (a research-use-only assay) incubated for 30 min at 55°C with gentle mixing and placed on the Abbott m2000sp instrument for sample extraction. The Abbott m2000sp/rt system used an open-mode protocol for DBS samples [20]. VL results were reported as “not detected,” if no HIV-1 RNA was detected in the sample. A qualitative result of “≤832 copies/mL (≤2.92 log copies)” was reported when fewer than or equal to 832 copies/mL of HIV-1 RNA were detected. Quantitative VL results were reported when HIV-1 RNA was detectable above 832 copies/mL (2.93 log copies to 7.00 log copies). A lower limit of quantification was not reported by the manufacturer as there is a low probability of reproducibility when samples have viremia ≤2.92 log copies (832 copies/mL). On the completion of DBS specimen analysis, aggregate results from the study were emailed to all consenting participants. We did not have IRB approval to provide individual results to participants.

### Survey Measures

The M-Spot survey was designed to assess the feasibility of collecting a DBS specimen for VL and also to capture HIV care information that may have occurred between the end of the parent study and enrollment in the M-Spot study. To reduce participant’s burden, HIV care questions were only asked if the participant reported seeing an HIV care provider after completing the 12-month survey (see Self-Reported Viral Load Status subsection). M-Spot survey data were merged with data from the parent study’s screener (herein referred to as *screener*) and the parent study’s 12-month follow-up survey (herein referred to as the *parent survey*). Demographic measures were primarily collected from the screener, HIV care and adherence measures for this analysis were collected from the parent survey, and DBS feasibility questions were collected from the M-Spot survey. Median time between the completion of the screener and the M-Spot survey was 405 days (range: 367-617 days), and median time between the completion of the parent survey and the M-Spot survey was 36 days (range: 6-257 days). All survey data were collected online.

#### Participant Characteristics

The screener included questions on participant’s age, race and ethnicity, gender identity, and sex at birth. Recruitment source was also identified from the screener, based on the recruitment URL used by the participant. Participants indicated on the parent survey whether they were diagnosed with HIV in the past year. The parent survey also obtained updated level of education, annual income, employment status, and insurance information.

#### Sexual History

Participants reported number of male anal insertive and receptive sex partners in the last 3 months on the parent survey. Pull-down menus listed 0 through 100 partners, 101+ partners, I don’t know, and prefer not to answer.

#### HIV Care

To assess engagement in HIV care, men were asked on the parent survey whether they had a doctor, nurse, or other medical provider whom they considered to be in charge of their overall HIV health care. Response options included no, yes, and prefer not to answer. Participants were also asked on the parent survey when was the last time they had a health care appointment with their HIV care provider (last 3 months, 3-6 months ago, 6-9 months ago, 9-12 months ago, more than a year ago, I don’t know, and prefer not to answer).

#### Antiretroviral Medication Adherence

Participants were asked on the parent survey about their current use of antiretroviral medications (yes, no). Among participants on treatment, past 30-day adherence to ART was assessed using a 3-item scale [18]. Participants were asked: “In the last 30 days, on how many days did you miss at least one dose of any of your HIV medicines?” (0-30 days); “In the last 30 days, how good a job did you do at taking your HIV medicines in the way you were supposed to?” (never, rarely, sometimes, usually, almost always, always); and “In the last 30 days, how often did you take your HIV medicines in the way you were supposed to?” (never, rarely, sometimes, usually, almost always, always). Responses to each question were linearly transformed to a 0 to 100 scale and averaged across all 3 items.

#### Self-Reported Viral Load Status

The M-Spot survey included items to measure self-reported VL status at the time of blood collection. Participants indicating an HIV care visit since the parent survey were asked whether they had a VL test. Men who reported having a VL test were asked to estimate the date of their last VL test and to select their most recent results from the following: My viral load was undetectable; My viral load was detectable; I don’t know—but I think I was detectable; and I don’t know—but I think I was undetectable. Participants reporting My viral load was detectable or I don’t know—but I think I was detectable were categorized as having a self-reported detectable VL status. Participants reporting My viral load was undetectable or I don’t know—but I think I was undetectable were categorized as having a self-reported undetectable VL status.

Data on self-reported VL status from participants who did not report an HIV care visit between the parent survey and the M-Spot study were obtained from the parent survey; men who reported a VL test in the past 6 months on the parent survey were asked to select their most recent results from the following: My viral load was undetectable, or <200 copies/mL; My viral load was detectable, or >200 copies/mL; I don’t know—but I think I was undetectable; and I don’t know—but I think I was detectable. Using the same strategy as in the M-spot survey, responses were dichotomized (detectable, undetectable). The date of the last VL test was not collected on the parent survey.

#### Time Between Self-Reported Viral Load and Dried Blood Spot Specimen Collection

The difference between the date of DBS specimen collection and date of self-reported VL status on the M-Spot or parent survey was used to estimate the time between a self-reported VL from a plasma sample (collected during an HIV care visit) and a VL laboratory result from a DBS specimen. The calendar date reported for last VL test in the M-Spot survey was used as the participant’s self-reported VL date. The parent survey did not ask participants to report the date of their last VL test. Thus, for participants who did not visit their HIV care provider in between the parent study and the M-Spot study, the day they finished the parent survey was used as a proxy for the participant’s self-reported VL test date. If participants did not self-report a VL status on either the M-Spot survey or parent survey, their self-reported VL status was treated as missing.

#### Experience Using Dried Blood Spot Kit

Experience using the DBS kit at home was measured through several questions on the M-Spot survey. Men were asked if they felt comfortable collecting their own blood sample (yes, no, prefer not to answer), and they were asked to rate their overall experience using the HemaSpot-HF device (very easy, easy, hard, very hard, prefer not to answer). Participants were also asked to rate their willingness to use a DBS kit in a future study (very willing, willing, not willing, extremely not willing, prefer not to answer).

### Statistical Methods

We assessed study feasibility by the proportion of participants who successfully completed various stages: enrollment, collecting a blood sample, mailing the kit to the laboratory, laboratory receipt of DBS specimens, and providing a testable blood sample. Pearson chi-square tests, Fisher exact tests, independent-sample *t*-tests, and Mann-Whitney *U* tests were used to identify group differences between participants who enrolled and did not enroll in the study and between participants who returned a DBS sample with detectable and undetectable viremia. Data analyses were performed using SAS version 9.4 (SAS Institute, Cary, NC).

## Results

### Participant Characteristics

**Figure 1:**
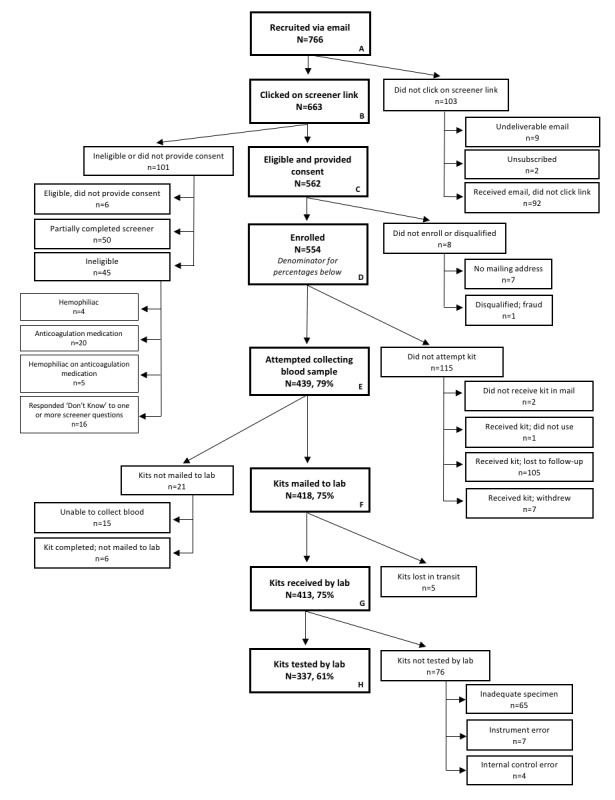
M-Spot study recruitment and participation. M-Spot: Mailed-Spot.

From September 2016 to February 2017, an invitation link to participate in the M-Spot study was sent to 766 men living with HIV within a week of completing the parent survey (Figure 1, box A). Of note, 112 men had completed the parent survey before we received IRB approval and thus were not eligible to participate in the study. Among recruited men, 86.6% (663/766) opened the email and clicked on the screener link (Figure 1, box B). Men who clicked the link were more likely to have health insurance (93.6% [617/659] vs 88.1% [89/101], *P*=.04) and a past 6-month HIV health care visit (92.4% [549/594] vs 83.0% [78/94], *P*<.01) than men who did not click the link. In total, 568 men were eligible to participate, 562 consented (Figure 1, box C), and 554 (72.3% [554/766] of those recruited) enrolled in the study (Figure 1, box D). To enroll, participants had to provide a mailing address to receive the DBS kit by mail.

Most enrolled participants were white (68.8%, 381/554), college-educated (61.7%, 341/553), and had a yearly income of less than US $40,000 (54.9%, 304/554; see Table 1). Median age was 39 years (range: 19-72 years). Most men (56.1%, 332/542) enrolled in M-Spot had been recruited for the parent study from a website for men interested in condomless anal sex with a male partner. Over half (57.8%, 320/554) were employed full time, and 94.0% (516/549) were insured—half through public health insurance. Participants self-reported a median of 2 male sexual partners (range: 0-101) in the past 3 months. A minority of men (19.1%, 105/551) were diagnosed with HIV in the 12 months before they enrolled in the parent study. On the basis of participants’ self-report, 91.1% (499/548) were engaged in HIV care; 93.0% (463/498) had visited their HIV care provider in the past 6 months; and 93.3% (516/553) were currently on ART, with a median Wilson adherence score of 88.9% (range: 0%-100%); and 90.8% (456/502) self-reported an undetectable VL (≤200 copies/mL) from their last clinical laboratory test. Compared with men who did not enroll in the M-Spot study, men who enrolled were more likely to have seen their HIV care provider in the past 6 months (93.0% [463/498] vs 86.3% [164/190], *P*=.02) and more likely to report ART use (93.3% [516/553] vs 89.1% [188/211], *P*=.05; see Table 1).

### Feasibility and Acceptability

Of the 554 men enrolled in M-Spot, 79.2% (439/554) reported attempting to collect their blood (Figure 1, box E). Some participants (n=49) requested a second DBS kit; reasons included difficulties collecting their blood or losing the kit. Of these men, 11 had issues drawing blood with the lancet and attributed this to callused fingertips. The initial lancet used for this study had an 18-gauge blade with a 2.3-mm penetration depth. In response to lancet-related issues, we sent the 11 participants, and all subsequently enrolled participants, lancets that had a 21-gauge needle and 2.8-mm penetration depth.

A high proportion (75.5%, 418/554) mailed a DBS specimen to the laboratory (Figure 1, box F). The laboratory received and evaluated 413 kits (Figure 1, box G). The median time between sample collection and specimen receipt was 4 days (range: 1-69 days). Among the kits received, 76 were not analyzed: 65 had an inadequate amount of blood and were deemed untestable, an instrument error occurred when processing 7 specimens, and an internal control error occurred when processing 4 specimens. In total, 337 kits had a sufficient amount of blood and were tested (Figure 1, box H).

Among men who returned a DBS specimen to the laboratory and completed the study survey, 89.8% (326/363) reported feeling comfortable collecting their blood; 83.6% (306/366) rated their experience using the DBS kit as “very easy” or “easy,” and 98.1% (357/364) reported willingness to use an at-home DBS kit in the future.

Among 115 participants who did not attempt to use the DBS kit, 105 men received the kit but did not participate (ie, were lost to follow-up), 7 men withdrew from the study, 2 men never received the kit, and 1 participant decided not to use the kit after opening the package. The men who were lost to follow-up or withdrew were predominantly white (69.6%, 80/115), college-educated (58.3%, 67/115), earned less than US $40,000 (57.1%, 64/112), and had a lower Wilson ART adherence score (81% vs 85%; *P*=.04) than men participating in study activities. Finally, 15 participants were unable to collect their blood and did not mail their kit.

**Table 1:** Sociodemographic and behavioral characteristics of recruited participants, by enrollment status (N=766).

Characteristics		Total (N=766)	Enrolled (n=554)	Ineligible or not enrolled (n=212)	*P* value
**Age in years (n=765)^a^, n (%)**					.10^b^
	18-29	155 (20.3)	111 (20.1)	44 (20.8)	
	30-39	233 (30.5)	181 (32.7)	52 (24.5)	
	40-49	221 (28.9)	159 (28.8)	62 (29.3)	
	50-59	126 (16.5)	84 (15.2)	42 (19.8)	
	≥ 60	30 (3.9)	18 (3.3)	12 (5.7)	
**Race (n=766), n (%)**					.31^b^
	Black	124 (16.2)	83 (15.0)	41 (19.3)	
	Hispanic	120 (15.7)	90 (16.3)	30 (14.2)	
	White	522 (68.2)	381 (68.8)	141 (66.5)	
**Education (n=763)^a^, n (%)**					.55^b^
	High school diploma or less	68 (8.9)	53 (9.6)	15 (7.1)	
	Some college	224 (29.4)	159 (28.8)	65 (31.0)	
	College graduate	309 (40.5)	228 (41.2)	81 (38.6)	
	Professional or graduate degree	162 (21.2)	113 (20.4)	49 (23.3)	
**Income (n=743)^a^, n (%)**					.21^b^
	<$20,000	225 (30.3)	173 (31.9)	52 (25.9)	
	$20,000-$39,999	182 (24.5)	131 (24.2)	51 (25.4)	
	$40,000-$59,999	137 (18.4)	102 (18.8)	35 (17.4)	
	$60,000-$99,999	111 (14.9)	80 (14.8)	31 (15.4)	
	≥$100,000	88 (11.8)	56 (10.3)	32 (15.9)	
**Insured (n=757)^a^, n (%)**					.41^b^
	Yes, private health insurance	349 (46.1)	257 (46.8)	92 (44.2)	
	Yes, public health insurance	357 (47.2)	259 (47.2)	98 (47.1)	
	No	51 (6.7)	33 (6.0)	18 (8.7)	
Employed full time (n=766), n (%)		443 (57.8)	320 (57.8)	123 (58.0)	.95^b^
**Recruitment source (n=764)^a^, n (%)**					.44^b^
	Mobile phone app	234 (30.6)	170 (30.7)	64 (30.3)	
	Bareback website	453 (53.3)	332 (60.0)	121 (57.4)	
	Other sites	77 (10.1)	51 (9.2)	26 (12.3)	
Engaged in HIV care (n=758)^a^, n (%)		689 (90.9)	499 (91.1)	190 (90.5)	.80^b^
Currently on antiretroviral therapy (n=764)^a^, n (%)		704 (92.2)	516 (93.3)	188 (89.1)	.05^c^
Past year HIV diagnosis (n=762)^a^, n (%)		151 (19.8)	105 (19.1)	46 (21.8)	.39^a^
**Last HIV care visit (n=688)^a^, n (%)**					.02^b,c^
	<6 months	627 (91.1)	463 (93.0)	164 (86.3)	
	6-12 months	51 (7.4)	29 (5.8)	22 (11.6)	
	>12 months	10 (1.5)	6 (1.2)	4 (2.1)	
**Self-reported HIV viral load status (n=673)^a^, n (%)**					.18^b^
	Undetectable	617 (91.7)	456 (90.8)	161 (94.5)	
	Detectable	56 (8.3)	46 (9.2)	10 (5.9)	
Antiretroviral therapy adherence score, mean (n=703)^a^		84.5	84.6	84.3	.88^d^
Number of male anal sex partners, last 3 months, mean (n=766)		18.3	21.2	10.5	.56^e^

^a^Denominators vary because of missing data.

^b^Pearson chi-square test.

^c^Statistical significance at level *P*≤.05.

^d^Independent-sample *t* test.

^e^Mann-Whitney *U* test.

### Viral Load Results

**Figure 2:**
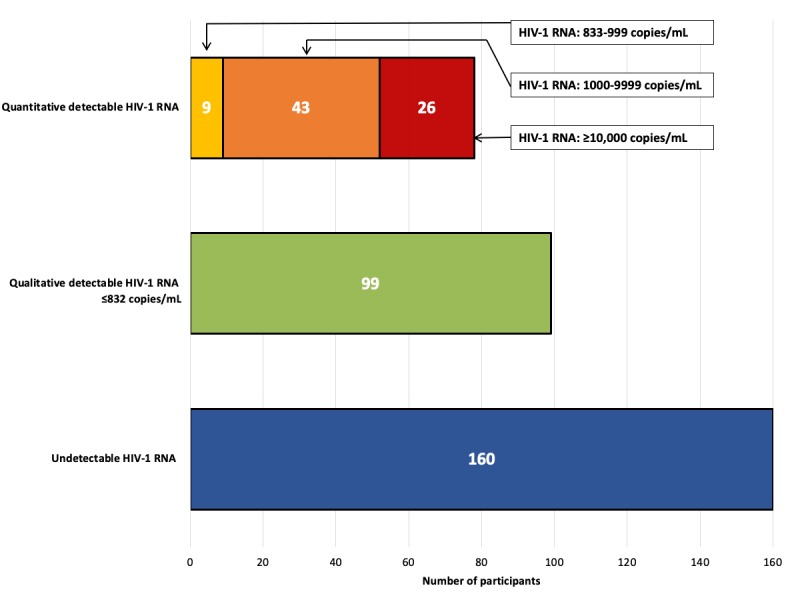
Participant laboratory HIV-1 RNA results, n=337.

Of the 337 specimens tested for VL by the laboratory, over half (52.5%, 177/337) had detectable VL, whereas 47.5% (160/337) of participants returned a sample with no detectable HIV-1 RNA (Figure 2). Of the DBS specimens classified as having a detectable VL, a total of 99 DBS specimens from participants had a qualitative result of “≤832 copies/mL (≤2.92 log copies),” and 78 DBS specimens had a quantitative result of >832 copies/mL. Among the DBS specimens with a quantitative VL (n=78), the overall median VL was 3508 copies/mL (interquartile range, IQR: 1349-21,754 copies/mL). When stratified into different levels of viremia, 9 specimens had detectable viremia between 833 and 999 copies/mL (median: 891 copies/mL; IQR: 870-955 copies/mL), 43 specimens were between 1000 and 9999 copies/mL (median: 1995 copies/mL; IQR: 1349-4542 copies/mL), and 26 specimens had viremia ≥10,000 copies/mL (median: 46,823 copies/mL; IQR: 22,264-144,865 copies/mL; see Figure 2). Compared with participants who returned a DBS specimen with detectable viremia, men who returned a DBS specimen with undetectable viremia were significantly more likely to be employed full-time (63.1% [101/160] vs 52.5% [93/177], *P*=.05), report a recent HIV diagnosis (<1 year; 23.1% [37/160] vs 12.5% [22/176], *P*=.01), be engaged in HIV care (96.9% [155/160] vs 86.4% [152/176], *P*<.01), and be currently on ART (98.8% [158/160] vs 88.6% [156/176], *P*<.01; see Table 2).

**Table 2:** Characteristics of participants who provided a testable dried blood spot specimen, by HIV-1 RNA viral load status (n=337).

Characteristics		Total (n=337)	Detectable HIV-1 RNA^a^, (n=177)	Undetectable HIV-1 RNA^a^, (n=160)	*P* value
**Age in years (n=337), n (%)**					.28^b^
	18-29	69 (20.5)	34 (19.2)	35 (21.9)	
	30-39	116 (34.4)	55 (31.1)	61 (38.1)	
	40-49	91 (27.0)	55 (31.1)	36 (22.5)	
	≥ 50	61 (18.1)	33 (18.6)	28 (17.5)	
**Race (n=337), n (%)**					.99^b^
	Black	41 (12.2)	22 (12.4)	19 (11.9)	
	Hispanic	57 (16.9)	30 (17.0)	27 (16.9)	
	White	239 (70.9)	125 (70.6)	114 (71.3)	
Employed full-time (n=337), n (%)		194 (54.6)	93 (52.5)	101 (63.1)	.05^b,c^
**Insured (n=333)^d^, n (%)**					.60^b^
	Yes, private health insurance	158 (47.5)	79 (44.9)	79 (50.3)	
	Yes, public health insurance	154 (46.3)	85 (48.3)	69 (44.0)	
	No	21 (6.3)	12 (6.8)	9 (5.7)	
Past year HIV diagnosis (n=335)^d^, n (%)		59 (17.6)	22 (12.5)	37 (23.3)	.01^b,c^
Engaged in HIV care (n=336)^d^, n (%)		307 (91.4)	152 (86.4)	155 (96.9)	.001^b,c^
**Last HIV care visit (n=307)^d^, n (%)**					.60^e^
	<6 months	286 (93.2)	140 (92.1)	146 (94.2)	
	6-12 months	17 (5.5)	9 (5.9)	8 (5.2)	
	>12 months	4 (1.3)	3 (2.0)	1 (0.7)	
**Self-reported HIV viral load status (n=316)^d^, n (%)**					<.001^b,c^
	Undetectable	284 (89.9)	135 (83.9)	149 (96.1)	
	Detectable	32 (10.1)	26 (16.2)	6 (3.9)	
Currently on ART^f^ (n=336)^d^, n (%)		314 (93.5)	156 (88.6)	158 (98.8)	<.001^b,c^
ART adherence score, mean (n=313)^d^		85.5	84.4	86.5	.33^g^
Number of male anal sex partners (mean), last 3 months (n=337)		26.5	29.8	22.9	.63^h^

^a^Individuals categorized as having a detectable HIV-1 RNA include participants with a quantitative result >832 copies/mL or qualitative result ≤832 copies/mL.

^b^Pearson chi-square test.

^c^Statistical significance at level *P*≤.05.

^d^Denominators vary because of missing data.

^e^Fisher exact test.

^f^ART: antiretroviral therapy.

^g^Independent-sample *t* test.

^h^Mann-Whitney *U* test.

**Figure 3:**
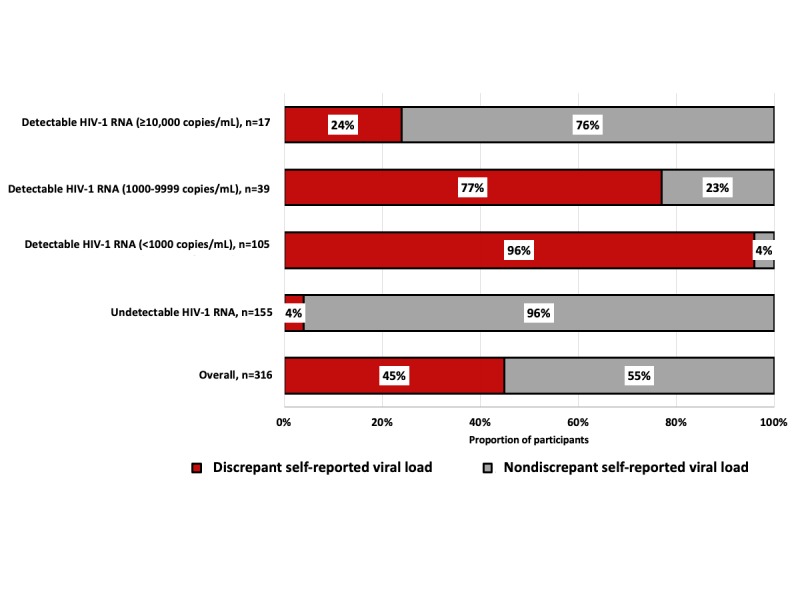
Participant self-reported viral load status, by laboratory HIV-1 RNA result, n=316.

We compared participants’ laboratory HIV-1 RNA result with their most recent self-reported VL. Among participants who returned a testable DBS specimen, 93.8% (316/337) also self-reported their VL in the M-Spot survey or the parent survey. Among the 316 DBS samples, 284 self-reported having an undetectable VL and 32 self-reported having a detectable VL. Among men with both laboratory and self-reported VL data, 44.6% (141/316) had a discrepant laboratory HIV-1 RNA result and self-reported VL status (Figure 3).

Of note, 83.9% (135/161) of the men who returned a DBS specimen with a detectable HIV-1 RNA result self-reported that they had an undetectable VL at their last clinical visit (Table 2). Among men self-reporting an undetectable VL, those living with HIV for >1 year at the start of the parent study were more likely to have a discrepant self-reported VL and a laboratory-quantified VL (88.2% [119/135] vs 77.0% [114/148]; *P*=.01). However, those who self-reported being engaged in care were less likely to have discrepancies between their self-reported VL and laboratory-quantified VL compared with those who self-reported not being engaged in care (91.1% [123/135] vs 97.3% [145/149]; *P*=.02).

Median time between a discrepant self-reported VL and a laboratory-quantified VL was 22 days. Median time between concordant self-reported VL and a laboratory-quantified VL was 25 days. Different proportions of discrepant self-reported VL were observed when the HIV-1 RNA results from the DBS specimens were disaggregated: 96.2% (101/105) of men with a laboratory HIV-1 RNA result <1000 copies/mL, 76.9% (30/39) of men with a laboratory HIV-1 RNA result <10,000, and 23.5% (4/17) of men with a laboratory HIV-1 RNA result ≥10,000 copies/mL had a discrepant self-reported VL (Figure 3 —indicated in red). Finally, an additional 21 participants returned a testable DBS specimen but did not self-report their VL in the survey. Of these men, 16 returned a sample with detectable viremia; of these, 13 men had a VL >1000 copies/mL (median: 20,893 copies/mL; range: 3467-154,881 copies/mL).

## Discussion

### Principal Findings

This study assessed the feasibility and acceptability of an at-home DBS collection kit for laboratory VL quantification from US MSM living with HIV who had previously reported suboptimal ART adherence or a detectable VL. To our knowledge, this is the first DBS home collection study from a Web-based sample of MSM living with HIV who mailed a DBS specimen to a laboratory for VL quantification. Feasibility was demonstrated at multiple study stages: 72.3% (554/766) of recruited men enrolled; 79.2% (439/554) of enrolled men attempted to collect a blood sample; 75.5% (418/554) mailed their DBS specimen to the laboratory and were received by the laboratory; and 60.8% (337/554) provided a testable blood sample. Among participants who returned a kit with a testable blood sample, 52.5% (177/337) had a detectable VL. Of significance, 83.9% (135/161) of DBS specimens with a detectable VL were from men who self-reported that they had an undetectable VL at their last clinical visit. These results suggest that at-home DBS collection for laboratory-quantified VL is both feasible and acceptable and may serve as a VL monitoring platform for MSM.

Our results support the findings of other studies with respect to the acceptability of self-collecting biologic specimens[21-23] and the willingness of MSM to self-collect blood samples for HIV testing [24-28]. The traditional DBS sampling using Guthrie cards or filter paper disks has been used for more than 40 years [29-31] and is now common in epidemiologic studies [32,33]. DBS sampling has been used in nonclinical settings for quantifying VL to identify acute and undiagnosed HIV infections [34,35]. However, DBS collection in nonclinical settings for VL quantification in known HIV-positive cohorts has largely been unexplored until now.

Results from 3 recent studies [36-38] prompted the Centers for Disease Control and Prevention and the National Institute of Allergy and Infectious Diseases to declare that people on ART who have an undetectable VL have no risk of transmitting the virus to HIV-negative partners [39,40]. To prevent further HIV transmission, individuals diagnosed with HIV must be engaged in care, take ART as prescribed, and achieve and maintain viral suppression [36,41]. The “Undetectable=Untransmittable” campaign has the potential to promote the benefits of HIV treatment, help alleviate stigma, and prevent further HIV transmission [42]. However, for the campaign to be effective, an individual’s perceived undetectable VL status must accurately match their actual VL status. The high proportion of individuals with a discrepant laboratory HIV-1 RNA result and self-reported VL status reported in this study indicates that men either incorrectly perceived or falsely reported their actual VL status. A recent study of young MSM and transgender women living with HIV [43] similarly reports discrepant self-reported VL survey data with laboratory-based and electronic medical record VL measurements; approximately a third of participants had discrepant laboratory-measured VL and a self-reported VL status. Our study reports a much higher proportion (84% vs 34%) of men with discrepant VL data with differences likely because of 2 disparate study populations with different age and racial and ethnic distributions and recruitment methodologies. We echo the authors’ concerns that discrepancies between self-reported and laboratory VL data have significant ramifications for continued HIV transmission, validity of epidemiological studies (eg, data misclassification), and the success of public health campaigns. Furthermore, inaccurate perception of one’s VL status may have potential consequences for partner-seeking behaviors among HIV-positive MSM who believe that they are undetectable when they are not.

Although most men returned a DBS specimen with a testable blood sample, some participants experienced issues collecting their blood at home. We estimate that about 15% to 20% of participants had difficulties with the lancets provided with the DBS kit based on our email and phone communication with the participants. In addition, some reported difficulties depositing blood drops into the middle of the application surface, which prevented them from getting enough blood on the absorbent paper. Participants experiencing issues often requested a second kit (where we provided a different lancet with a deeper penetration depth), did not return their kit to the laboratory, or returned a kit to the laboratory with little or no blood. Future studies should anticipate possible specimen self-collection issues or issues with study materials such as lancets. Addressing these issues will likely increase the feasibility of at-home specimen collection.

Novel interventions, devices (eg, Food and Drug Administration–approved home VL test), and service delivery options (eg, sharing DBS VL laboratory results with a patient’s provider) must be developed to increase the number of individuals who are retained in care and who achieve and accurately perceive their current VL status. An individual’s VL is dynamic and viral “blips” can occur throughout the long-term treatment of HIV because of fluctuations in ART adherence or concurrent illnesses [44-47]. Individuals with a history of intermittent HIV care or detectable viremia may be more inclined to reengage in care or improve ART adherence, if they know they have a detectable (laboratory-quantified) VL from an at-home self-collected specimen; future studies should assess this, as well as whether there are clinical benefits with more frequent VL monitoring in between clinical visits from samples collected outside of clinical settings.

### Limitations

A few study limitations should be acknowledged. First, with respect to our email recruitment approach, it is possible that some participants never saw the email in their inbox or spam folder. Second, 20.8% (115/554) of enrollees did not complete study activities; participants who withdrew or were lost to follow-up had a lower Wilson ART adherence score than those who attempted or completed study activities. It is possible that these participants chose not to collect their blood sample after reading the instructions, or they never opened the DBS package with study materials. It is also possible that participants who reported suboptimal ART adherence did not want to provide a blood sample that would show a detectable VL. Third, the study population was recruited from a sample of men who successfully completed a 12-month Web-based intervention. Perhaps these participants are more likely to complete a study such as M-Spot compared with other populations. Different study participation rates may be observed when collecting DBS specimens from other populations. Fourth, we did not collect a calendar date for the most recent VL test on the parent survey, limiting our ability to accurately estimate the time between an individual’s plasma VL test result and DBS VL test result.

As with most DBS studies, there is concern about the correlation between a DBS specimen and laboratory result from a VL test from a plasma sample collected in a clinical setting. Several studies [48-50] have documented the high correlation between VL measurements obtained from DBS samples and those obtained from plasma, as well as the stability of samples stored under different conditions and for different time frames. In addition, stability of HIV serological markers in samples collected using the HemaSpot device has been reported and compared with DBS samples collected using the traditional Whatman 903 cards [51]. Similarly, studies assessing the performance of the Abbott Real-Time HIV-1 assay, used for this study, have also reported a high correlation between VL measurements from DBS and plasma samples, with 99.4% of cases differing by <1.0 log copies in one study [52], and a mean difference of 0.29 log copies in another [53]. The Abbott assay and extraction method have been updated and improved frequently; this study used the most up-to-date open mode protocol available from the manufacturer. Dize et al performed validation of DBS specimens collected using HemaSpot devices compared with plasma samples [54]. Concordance analysis showed 100% agreement between samples with VL ≥1000 copies/mL and 86% agreement between samples with VL <1000 copies/mL. Finally, we acknowledge the possibility that intracellular nucleic acid contribution at low levels of viremia may explain our DBS specimen laboratory results and the discordance between self-reported VL status and DBS specimen VL results. Other studies have reported false-positive rates ranging from 6% to 13% in which DBS samples yielded detectable VL, whereas the plasma sample had undetectable VL, with the discrepancy attributed to a possible contribution of intracellular RNA that might be present in white blood cells in whole blood [55-58]. However, it is unlikely that intracellular nucleic acid led to misclassification of VL results for participants who had high HIV VL results from their DBS specimen. Further research is needed to identify the extent to which intracellular nucleic acid influences VL results from DBS specimens collected using the HemaSpot-HF device, especially in those with low detectable HIV VL levels.

### Conclusions

Despite these limitations, findings from this study highlight the feasibility and acceptability of HIV-1 RNA quantification of home-collected DBS samples from MSM living with HIV. Individuals with a history of suboptimal ART adherence and/or detectable viremia, such as those in this study, may benefit from at-home VL monitoring as a tool to augment engagement in HIV care. Home collection of DBS for VL could be utilized as a monitoring tool in between clinical visits for patients who struggle with adherence. Research on complementary systems of clinical care should be expanded and further studied, especially in the era of treatment as prevention.

## Abbreviations

ART: antiretroviral therapy
DBS: dried blood spot
DBS kit: HemaSpot-HF device
IRB: institutional review board
IQR: interquartile range
M-Spot: Mailed-Spot
MSM: men who have sex with men
VL: HIV-1 RNA viral load
